# Overexpression of *TaMYB4* Confers Freezing Tolerance in *Arabidopsis thaliana*

**DOI:** 10.3390/ijms241311090

**Published:** 2023-07-04

**Authors:** Yu Tian, Kankan Peng, Xuan Ma, Zhipeng Ren, Guicheng Lou, Yunshuang Jiang, Jingqiu Xia, Duojia Wang, Jing Yu, Jing Cang

**Affiliations:** College of Life Science, Northeast Agricultural University, Harbin 150030, China

**Keywords:** TaMYB4, winter wheat, freezing stress, AsA–GSH cycle, DREB/CBF signaling

## Abstract

Freezing stress is one of the main factors limiting the growth and yield of wheat. In this study, we found that *TaMYB4* expression was significantly upregulated in the tillering nodes of the strong cold-resistant winter wheat variety Dongnongdongmai1 (Dn1) under freezing stress. Weighted gene co-expression network analysis, qRT-PCR and protein–DNA interaction experiments demonstrated that monodehydroascorbate reductase (*TaMDHAR*) is a direct target of TaMYB4. The results showed that overexpression of *TaMYB4* enhanced the freezing tolerance of transgenic *Arabidopsis*. In *TaMYB4* overexpression lines (OE-*TaMYB4*), *AtMDHAR2* expression was upregulated and ascorbate-glutathione (AsA–GSH) cycle operation was enhanced. In addition, the expression of cold stress marker genes such as *AtCBF1*, *AtCBF2*, *AtCBF3*, *AtCOR15A*, *AtCOR47*, *AtKIN1* and *AtRD29A* in OE-*TaMYB4* lines was significantly upregulated. Therefore, TaMYB4 may increase freezing tolerance as a transcription factor (TF) in *Arabidopsis* through the AsA–GSH cycle and DREB/CBF signaling pathway. This study provides a potential gene for molecular breeding against freezing stress.

## 1. Introduction

Wheat (*Triticum aestivum*) is the second most cultivated cereal crop worldwide. The increased human population combined with modern consumption preferences have led to a proliferation in the demand for wheat over the past 50 years [1]. However, various abiotic stresses severely limit wheat production [2]. Among them, cold stress mainly limits wheat growth and geographic distribution, and affects wheat yield and quality [3]. The winter season is long and cold in the Heilongjiang Province of China. Dongnongdongmai1 (Dn1) is the first winter wheat breed to be successfully overwintered in Heilongjiang Province [4]. A large amount of physiological indices and gene expression data reflect the distinct cold-resistance characteristics of Dn1 [5,6,7]. Nevertheless, the regulatory mechanism of transcription factors (TFs) in Dn1 under cold stress is still poorly understood.

The MYB TF family contributes prominently in terms of plant responses to abiotic stresses [8]. In apple (*Malus pumila*), MdMYB108L and MdHY5 shape a feedback circuit to regulate *CBF3* expression, and overexpression of *MdMYB108L* improves apple cold tolerance [9]. In *Arabidopsis thaliana*, the MYB96-HHP module activates the CBF pathway involved in regulating cold tolerance in the plants [10]. In maize (*Zea mays*), *ZmMYB31* expression is upregulated under cold stress, and overexpression of *ZmMYB31* elevates SOD and APX activities in *Arabidopsis* [11]. In rice (*Oryza sativa*), the regulatory network centered on OsMYB4 enhances cellular antioxidant capacity under cold stress [12]. In wheat, 60 *MYB* genes have been isolated, of which, 11 gene expressions are affected by cold stress [13]. Recently, we screened *TaMYB4* in Dn1, which can respond to freezing stress [14]. Nevertheless, the regulatory mechanism of MYB TFs in wheat under freezing stress is still poorly understood.

Monodehydroascorbate reductase (MDHAR, EC 1.6.5.4) is a momentous enzyme in the ascorbate–glutathione (AsA–GSH) cycle that catalyzes the regeneration of AsA from MDHA [15]. Studies have shown that the transcription and activity of MDHAR are significantly upregulated in tomato (*Solanum lycopersicum*) [16], acerola (*Malpighia glabra*) [17] and Antarctic hairgrass (*Deschampsia antarctica*) [18] under cold stress. Overexpression of *Brassica rapa MDHAR* enhances the antioxidant capacity and improves freezing tolerance in *Arabidopsis* [19]. Furthermore, the co-expression of *BrDHAR* and *BrMDHAR* produces synergistic effects and is also able to effectively improve the freezing tolerance of *Arabidopsis* [20]. In wheat, MDHAR transcription and activity are significantly upregulated under drought, heat and water stress [21,22,23]. However, the mechanism of transcriptional regulation of *MDHAR* under freezing stress remains unknown.

In this research, we discovered that under either severe winter field or short-term indoor freezing treatment, *TaMYB4* expression in Dn1 was significantly upregulated. Protein–DNA interaction experiments demonstrated that *TaMDHAR* is a direct target of TaMYB4. Overexpression of *TaMYB4* or *TaMDHAR* promoted the AsA–GSH cycle operation and improved *Arabidopsis* freezing tolerance. The in-depth exploration of the transcriptional regulation mechanism of *MDHAR* by TaMYB4 not only enriches the TaMYB4 regulatory network, but also provides a theoretical basis for crop cold-resistance breeding.

## 2. Results

### 2.1. TaMYB4 Is Induced by Cold

**Figure 1:**
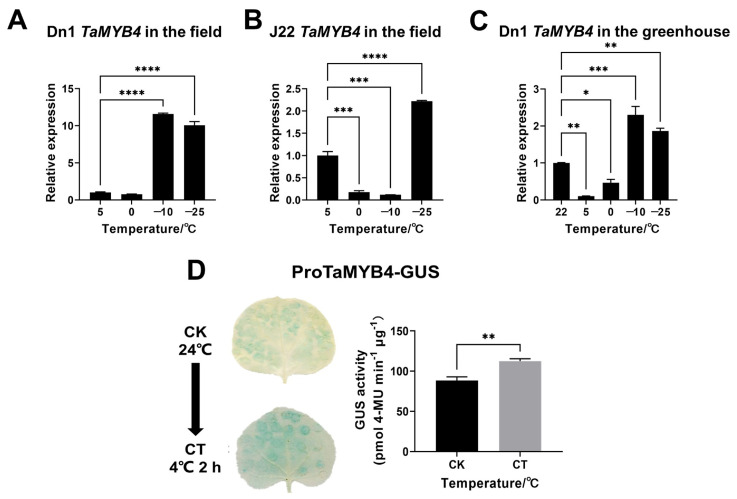
*TaMYB4* expression analysis in tillering nodes of winter wheat and *TaMYB4* promoter activity analysis under cold stress. (**A**,**B**) *TaMYB4* expression in field-grown Dn1 and J22; (**C**) *TaMYB4* expression in greenhouse-grown Dn1; (**D**) Promoter activity analysis of *TaMYB4*. Mean ± SD (n = 3) is used to represent values. Significant differences in panels (**A**–**C**) were calculated by one-way ANOVA and marked by asterisks; Significant differences in panel (**D**) were calculated by Student’s *t*-test and marked by asterisks; *, *p* < 0.05; **, *p* < 0.01; ***, *p* < 0.001; ****, *p* < 0.0001. CK: control check, CT: cold treatment.

Previously, we found that *TaMYB4* expression was significantly upregulated in the tillering nodes of Dn1 under freezing stress by transcriptome analysis [14]. To experimentally demonstrate the cold induction of *TaMYB4* in the transcriptome data, we detected *TaMYB4* expression in the tillering nodes of field-grown Dn1 by qRT-PCR (5 °C as a control). The expression of *TaMYB4* in Dn1 at −10 and −25 °C was significantly higher than that at 5 °C, with fold changes of 11.60 and 11.06, respectively (Figure 1A). However, there was only a two-fold increase in the expression of *TaMYB4* at −25 °C in Jimai22 (J22) (Figure 1B). To exclude the interference of spatiotemporal differences and other environmental factors in the field on the expression of *TaMYB4*, we subjected Dn1 seedlings to short-term low-temperature treatment and performed qRT-PCR analysis (22 °C as a control). The *TaMYB4* expression pattern in the tillering nodes of greenhouse-grown Dn1 was similar to that of field-grown Dn1, and *TaMYB4* expression was significantly upregulated at −10 and −25 °C (Figure 1C).

Then, we performed a promoter activity assay under cold stress. The promoter of *TaMYB4* (ProTaMYB4) contained a low-temperature-responsive element (Figure S1). The ProTaMYB4 activity was detected using the GUS reporter gene in tobacco leaves transiently transfected with pBI121-ProTaMYB4-GUS. We observed that the blue color of tobacco leaves deepened after cold treatment at 4 °C. After cold stress, the GUS activity of the tobacco leaves transiently transfected with pBI121-ProTaMYB4-GUS was also markedly higher than in leaves without cold stress (Figure 1D). This suggests that ProTaMYB4 is induced by cold temperatures.

### 2.2. TaMYB4 Co-Expression Network Construction

**Figure 2:**
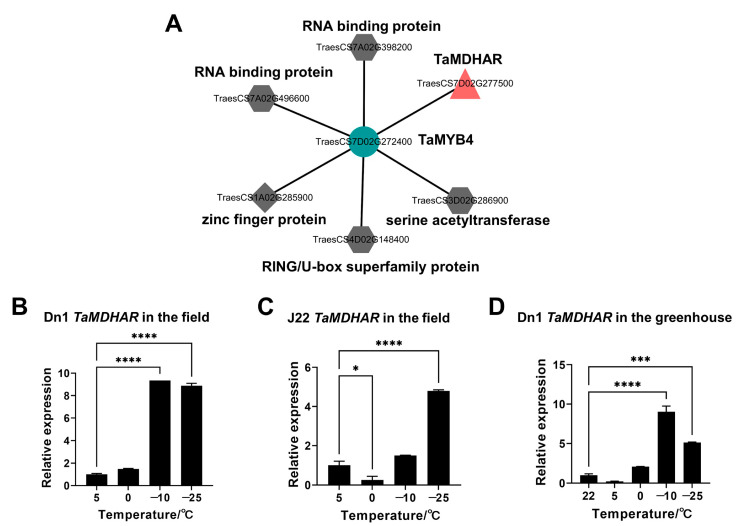
*TaMYB4* co-expression network and *TaMDHAR* expression analysis in tillering nodes of winter wheat under cold stress. (**A**) *TaMYB4* co-expression network; (**B**,**C**) *TaMDHAR* expression in field-grown Dn1 and J22; (**D**) *TaMDHAR* expression in greenhouse-grown Dn1. Mean ± SD (n = 3) is used to represent values. One-way ANOVA was used to calculate significant differences, which are marked by asterisks; *, *p* < 0.05; ***, *p* < 0.001; ****, *p* < 0.0001.

To explore the TaMYB4 regulatory network under freezing stress, weighted gene co-expression analysis was performed. We found that six members were associated with TaMYB4 (Figure 2A). These members included zinc finger protein (TraesCS1A02G285900), serine acetyltransferase (TraesCS3D02G286900), RING/U-box superfamily protein (TraesCS4D02G148400), RNA binding protein (TraesCS7A02G398200), kelch-type beta propeller domain containing protein (TraesCS7A02G496600) and monodehydroascorbate reductase (TraesCS7D02G277500). We observed that monodehydroascorbate reductase (MDHAR) gene expression was significantly upregulated in the Dn1 transcriptome under freezing stress, and that its expression was the highest among the six members (Figure S2).

Then, we detected *TaMDHAR* expression in the tillering nodes of field-grown winter wheat. *TaMDHAR* expression in Dn1 was significantly increased at −10 °C and −25 °C, and the fold change was higher than that in J22 (Figure 2B,C). To exclude the interference of spatiotemporal differences and other environmental factors in the field on the expression of *TaMDAHR*, we subjected Dn1 seedlings to short-term low-temperature treatment and performed qRT-PCR analysis (22 °C as a control). The *TaMDHAR* expression pattern of greenhouse-grown Dn1 was similar to that of field-grown Dn1 (Figure 2D). We found that *TaMDHAR* had a similar expression pattern to *TaMYB4* in Dn1 under freezing stress. Therefore, we speculated that there might be an association between *TaMYB4* and *TaMDHAR* under freezing stress.

### 2.3. Bioinformatics Analysis and Subcellular Localization of TaMYB4 and TaMDHAR

We cloned *TaMYB4* and *TaMDHAR* sequences from Dn1 cDNA. Multiple sequence alignment showed that TaMYB4 contained two typical conserved adjacent repeats in the MYB domain and belongs to the R2R3 subgroup (Figure S3A). The phylogenetic tree that was generated showed that TaMYB4 clustered with TaMYB-1D and ZmMYB31 (Figure S3B). Multiple sequence alignments revealed that TaMDHAR is highly similar to MDHAR in other plants and contains a typical Pyr_redox_2 domain (Figure S3C). The phylogenetic tree constructed from these proteins showed that TaMDHAR clustered with AeMDHAR (Figure S3D).

**Figure 3:**
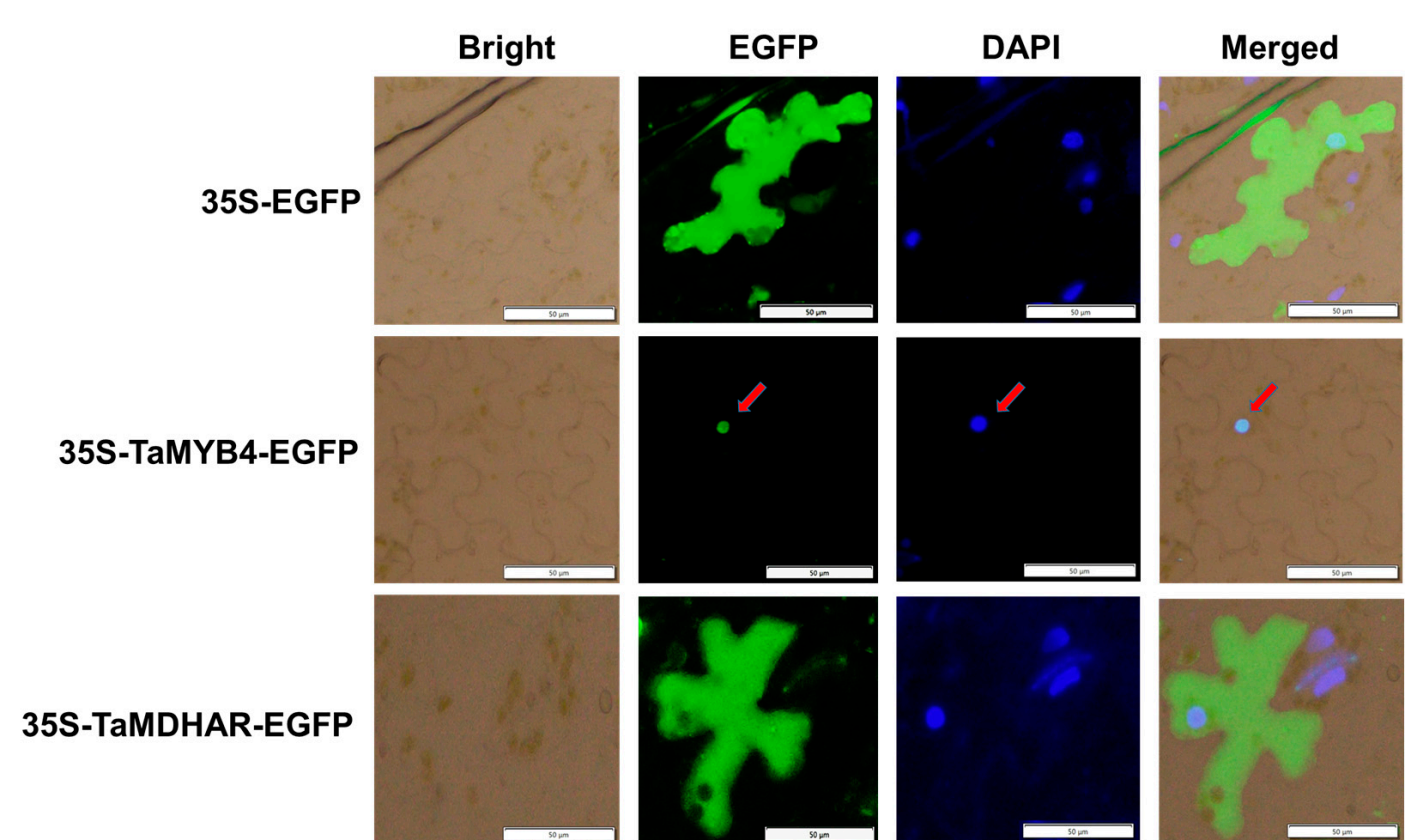
Subcellular localization analysis of TaMYB4 and TaMDHAR in tobacco leaves. 35S-EGFP: tobacco expressing 35S-EGFP (vector control); DAPI (4′, 6-diamidino-2-phenylindole): A blue fluorescent DNA dye used to mark the location of the nucleus; Red arrow: point to the location of the nucleus.

**Figure 4:**
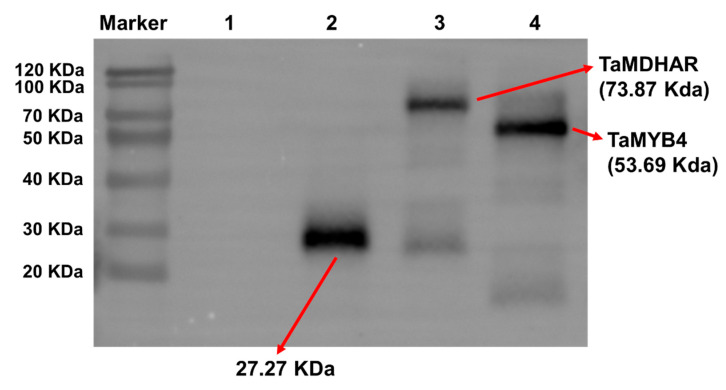
Western blot analysis of TaMYB4 and TaMDHAR in tobacco leaves. Lane 1: Total protein extracted from un-injected tobacco; Lane 2: Total protein extracted from tobacco expressing 35S-EGFP; Lane 3: Cytosolic protein extracted from tobacco expressing TaMDHAR-EGFP; Lane 4: Nuclear protein extracted from tobacco expressing TaMYB4-EGFP; Red arrows: mark the positions of different proteins.

Subcellular localization prediction analysis revealed that TaMYB4 might localize to the nucleus, whereas TaMDHAR might localize to the cytoplasm (Figure S3E,F). Subsequently, we found that the enhanced green fluorescent protein (EGFP) fluorescence signal was mainly detected in the nucleus in tobacco leaves containing the TaMYB4-EGFP plasmid. And the EGFP fluorescence signal was mainly detected in the cytoplasm in tobacco leaves containing the TaMDHAR-EGFP plasmid (Figure 3). To further determine the localization of TaMYB4 and TaMDHAR, we isolated nuclear and cytosolic proteins from tobacco leaves expressing TaMYB4-EGFP and TaMDHAR-EGFP, respectively. Then, we performed Western blot analysis. The results showed that TaMYB4 and TaMDHAR were detected in the nucleus and cytoplasm using anti-EGFP antibody, respectively (Figure 4).

### 2.4. TaMYB4 Regulates TaMDHAR Expression under Cold Stress

**Figure 5:**
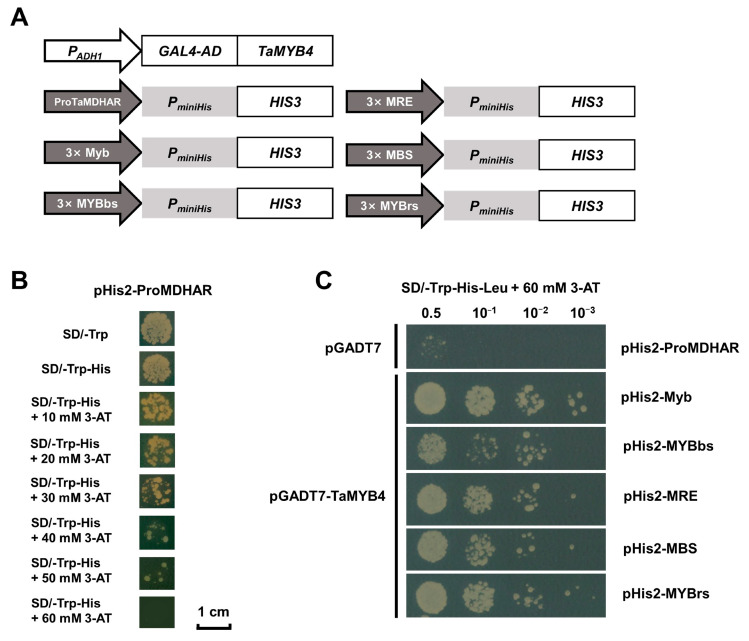
Yeast one-hybrid validation of the regulatory relationship between TaMYB4 and TaMDHAR. (**A**) Schematic diagram of different constructs used in yeast one-hybrid assays; (**B**) Autoactivity test of the pHis2-ProMDHAR vector; (**C**) TaMYB4 binds to the MYB binding site in vivo. The pGADT7 empty and pHis2-ProMDHAR vectors were co-transferred into yeast as a control.

Eight MYB binding sites were present in the isolated 2000 bp promoter of *TaMDHAR* (ProTaMDHAR) (Figure S1B). *TaMDHAR* may be a direct target of TaMYB4. To validate this regulatory relationship, a yeast one-hybrid assay was performed. The eight MYB binding sites in ProTaMDHAR were classified into five categories, including MRE (AACCTAA), MBS (CAACTG), Myb (CAACAG), MYBrs (CCGTTG) and MYBbs (TAACCA). Full-length ProTaMDHAR and three tandem copies of the motif sequence were cloned into the pHis2 vector. Then, the yeast strain Y187 was co-transformed by the pHis2 recombinant plasmid with the pGADT7-TaMYB4 recombinant plasmid. We found that TaMYB4 can recognize five MYB binding sites and has stronger binding activity to the Myb motif (Figure 5).

**Figure 6:**
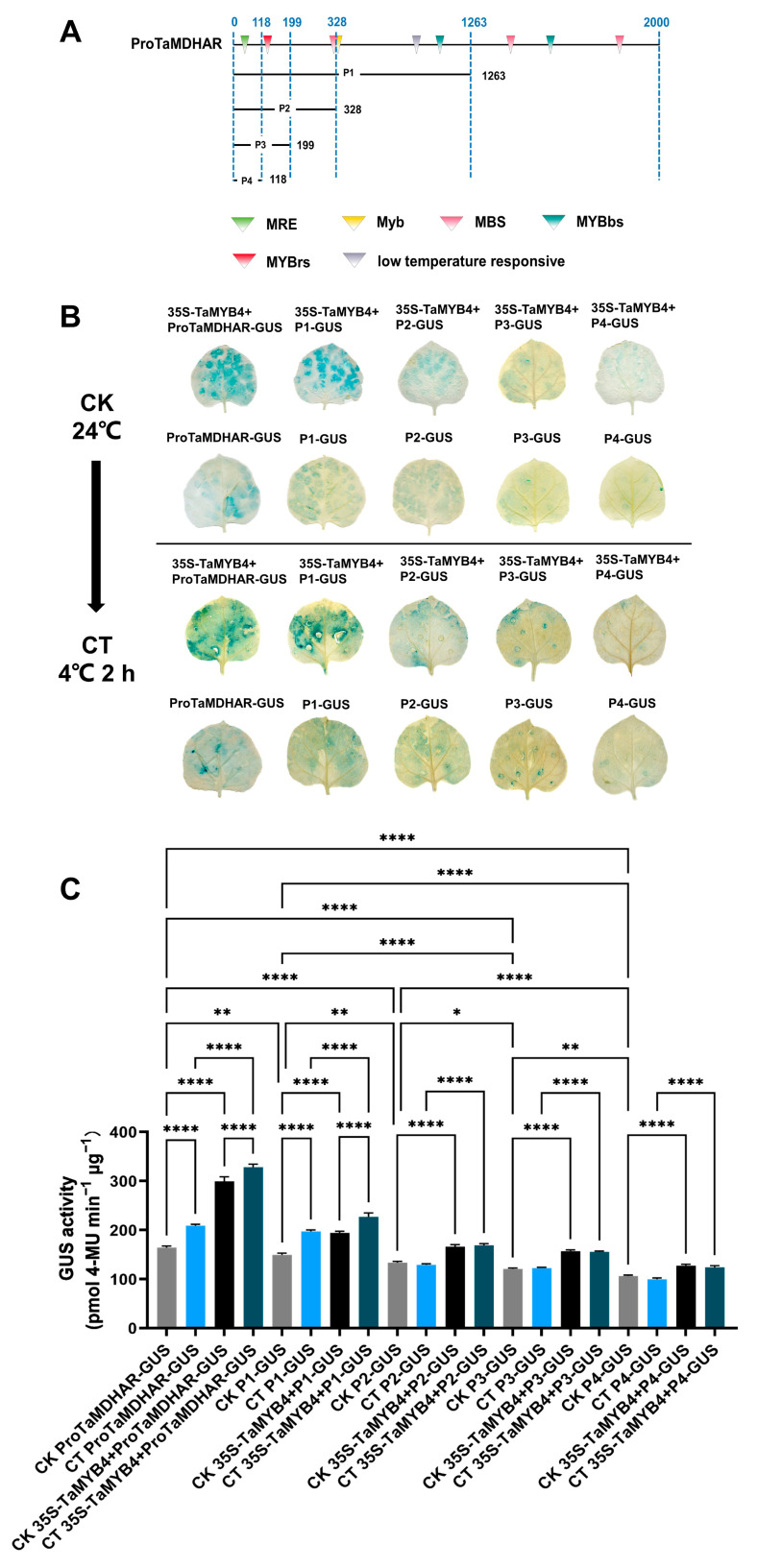
GUS transient expression experiment validation of the regulatory relationship between TaMYB4 and *TaMDHAR*. (**A**) Schematic diagram of ProTaMDHAR truncation; (**B**) GUS staining of tobacco leaves; (**C**) GUS activity in tobacco leaves. Mean ± SD (n = 3) is used to represent values. One-way ANOVA was used to calculate significant differences, which are marked by asterisks; *, *p* < 0.05; **, *p* < 0.01; ****, *p* < 0.0001. CK: control check, CT: cold treatment.

Subsequently, we performed a GUS transient expression experiment. We truncated ProTaMDHAR according to the distribution of the MYB binding sites (Figure 6A). The full-length and truncated sequences of ProTaMDHAR were cloned into the pBI121 vector. Then, tobacco leaves were transiently transfected with PBI121 recombinant plasmid alone or together with 35S-TaMYB4 recombinant plasmid. After 3 d of culture, the tobacco was divided into control and cold-treated (4 °C 2 h) groups. The GUS staining results showed that the blue color of the tobacco leaves gradually became lighter with shortening of the promoter sequence. The P1 and ProMDHAR sequences presented a low-temperature-responsive element. The color of tobacco leaves transiently expressing pBI121-ProTaMDHAR-GUS and pBI121-P1-GUS deepened under cold stress. Moreover, the addition of the 35S-TaMYB4 recombinant plasmid noticeably deepened the color of the tobacco leaves (Figure 6B). We subsequently assayed GUS activity in tobacco leaves, and the GUS activity change was in line with the tobacco leaf color change (Figure 6C).

**Figure 7:**
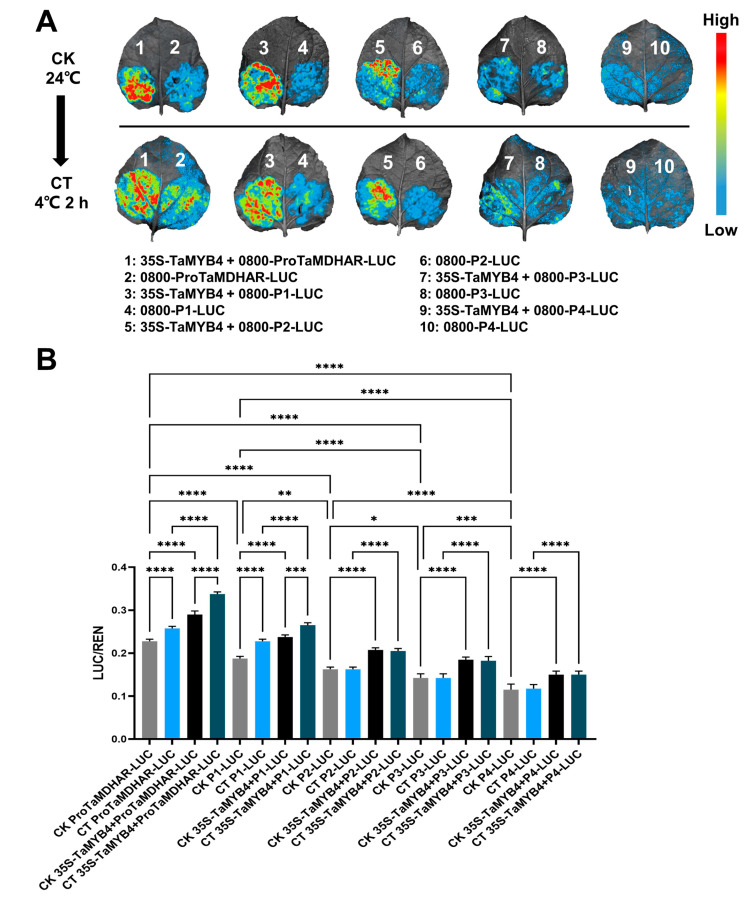
Dual luciferase transient expression experiment validation of the regulatory relationship between TaMYB4 and *TaMDHAR*. (**A**) LUC signal detected in tobacco leaves; (**B**) Relative LUC/REN ratio in tobacco leaves. Mean ± SD (n = 3) is used to represent values. One-way ANOVA was used to calculate significant differences, which are marked by asterisks; *, *p* < 0.05; **, *p* < 0.01; ***, *p* < 0.001; ****, *p* < 0.0001. CK: control check, CT: cold treatment.

The results of dual luciferase transient expression experiments were similar to those of the GUS transient expression experiments. The LUC/REN in the tobacco leaves decreased gradually with the shortening of the promoter sequence, and the LUC/REN in tobacco leaves transiently expressing 0800-ProTaMDHAR-LUC and 0800-P1-LUC was significantly increased under cold stress. Moreover, the addition of the 35S-TaMYB4 recombinant plasmid significantly increased the LUC/REN ratio in the tobacco leaves (Figure 7).

### 2.5. Characterization of TaMYB4 and TaMDHAR Function under Freezing Stress

**Figure 8:**
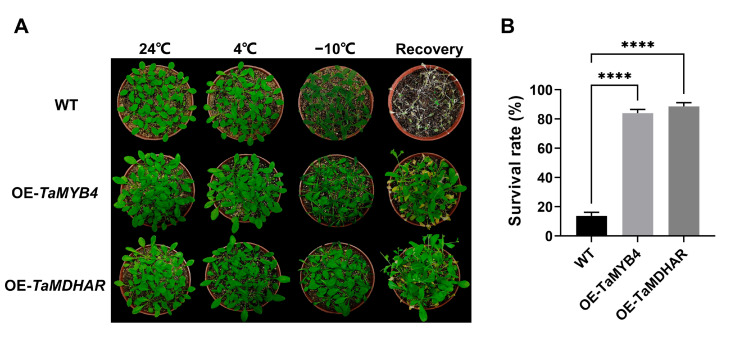
Phenotype observation and survival rate statistics of the OE-*TaMYB4* and OE-*TaMDHAR* lines under freezing stress. (**A**) Phenotype observation; (**B**) Survival rate statistics. Mean ± SD (n = 3) is used to represent values. Student’s *t*-test was used to calculate significant differences, which are marked by asterisks; ****, *p* < 0.0001.

**Figure 9:**
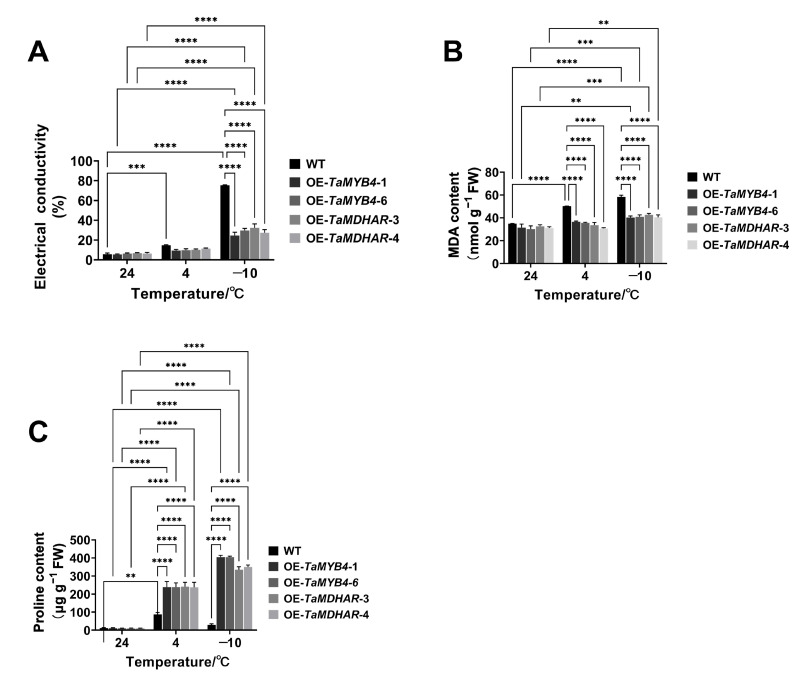
Detection of physiological indices of the OE-*TaMYB4* and OE-*TaMDHAR* lines under freezing stress. (**A**) Electrical conductivity; (**B**) MDA content; (**C**) Pro content. Mean ± SD (n = 3) is used to represent values. Two-way ANOVA was used to calculate significant differences, which are marked by asterisks; **, *p* < 0.01; ***, *p* < 0.001; ****, *p* < 0.0001.

To characterize the functions of *TaMYB4* and *TaMDHAR* under freezing stress, we constructed expression vectors (35S-TaMYB4 and 35S-TaMDHAR) and transformed them into *Arabidopsis*. After several cycles of kanamycin-resistance screening, *TaMYB4* and *TaMDHAR* overexpression lines (OE-*TaMYB4*-1, OE-*TaMYB4*-6, OE-*TaMDHAR*-3 and OE-*TaMDHAR*-4) were obtained. We observed the phenotypes of the *Arabidopsis* lines (WT, OE-*TaMYB4* and OE-*TaMDHAR*) under freezing stress (Figure 8A). After −10 °C treatment, curling and a deepening in color occurred in the leaves of the three lines, but the WT had a deeper leaf color than the OE-*TaMYB4* and OE-*TaMDHAR* lines. After recovery for 7 d, the OE-*TaMYB4* and OE-*TaMDHAR* lines mostly recovered to a normal growth state, whereas nearly all of the WT died. Ultimately, the OE-*TaMYB4* and OE-*TaMDHAR* lines had higher survival rates than WT (Figure 8B). Then, we determined the electrical conductivity (EC), malondialdehyde (MDA) and proline (Pro) contents. The results showed that the OE-*TaMYB4* and OE-*TaMDHAR* lines had a lower EC and MDA content and a higher Pro content than WT at 4 °C and −10 °C. Altogether, the OE-*TaMYB4* and OE-*TaMDHAR* lines showed equally excellent physiological properties under freezing stress (Figure 9).

**Figure 10:**
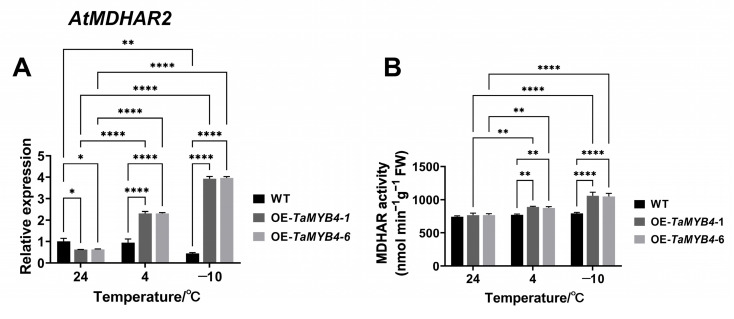
Expression of *AtMDHAR2* (**A**) (24 °C WT as a control) and activity of MDHAR (**B**) in the OE-*TaMYB4* lines. Mean ± SD (n = 3) is used to represent values. Two-way ANOVA was used to calculate significant differences, which are marked by asterisks; *, *p* < 0.05; **, *p* < 0.01; ****, *p* < 0.0001.

TaMDHAR had high homology with *Arabidopsis* cytoplasmic subtype AtMDHAR2 (Figure S3D). MYB binding sites were present in the *AtMDHAR2* promoter (Figure S4). OE-*TaMYB4* lines showed higher *AtMDHAR2* expression and MDHAR activity than WT at 4 °C and −10 °C (Figure 10).

### 2.6. ROS Scavenging Capacity Is Increased in OE-TaMYB4 and OE-TaMDHAR Arabidopsis under Freezing Stress

**Figure 11:**
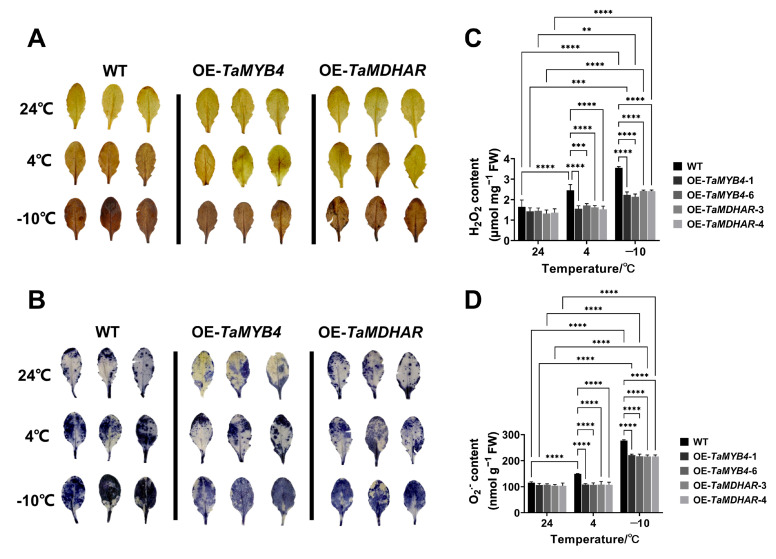
Detection of ROS content in the OE-*TaMYB4* and OE-*TaMDHAR* lines under freezing stress. DAB (**A**) and NBT (**B**) staining of *Arabidopsis* leaves; H_2_O_2_ (**C**) and O_2_·^–^ (**D**) contents in *Arabidopsis* leaves. Mean ± SD (n = 3) is used to represent values. Two-way ANOVA was used to calculate significant differences, which are marked by asterisks; **, *p* < 0.01; ***, *p* < 0.001; ****, *p* < 0.0001.

To demonstrate that overexpression of *TaMYB4* and *TaMDHAR* confers stronger ROS scavenging capacity in *Arabidopsis*, we performed the 3,3′-diaminobenzidine (DAB) and nitroblue tetrazolium (NBT) staining. We found that the WT had a deeper leaf color than the OE-*TaMYB4* and OE-*TaMDHAR* lines under freezing stress (Figure 11A,B). To further demonstrate the ROS changes in the WT, OE-*TaMYB4* and OE-*TaMDHAR* lines under freezing stress, we determined the hydrogen peroxide (H_2_O_2_) and superoxide anion (O_2_^•−^) contents, which were higher in the WT than those in the OE-*TaMYB4* and OE-*TaMDHAR* lines under freezing stress (Figure 11C,D).

### 2.7. The AsA–GSH Cycle Is Improved in OE-TaMYB4 and OE-TaMDHAR Arabidopsis under Freezing Stress

**Figure 12:**
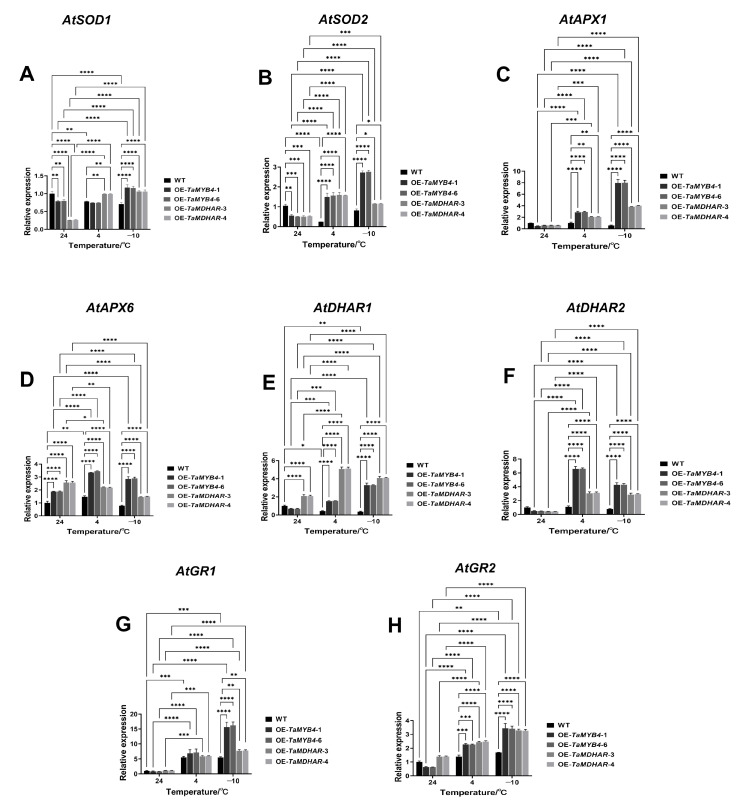
Expression of *AtSOD1* (**A**), *AtSOD2* (**B**), *AtAPX1* (**C**), *AtAPX6* (**D**), *AtDHAR1* (**E**), *AtDHAR2* (**F**), *AtGR1* (**G**) and *AtGR2* (**H**) in the OE-*TaMYB4* and OE-*TaMDHAR* lines under freezing stress. Mean ± SD (n = 3) is used to represent values. Two-way ANOVA was used to calculate significant differences, which are marked by asterisks (24 °C WT as a control); *, *p* < 0.05; **, *p* < 0.01; ***, *p* < 0.001; ****, *p* < 0.0001.

**Figure 13:**
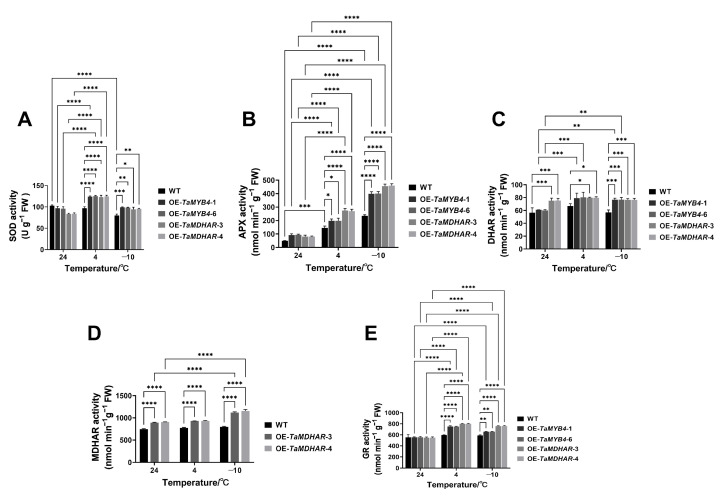
Activities of SOD (**A**), APX (**B**), DHAR (**C**), MDHAR (**D**) and GR (**E**) activity in the OE-*TaMYB4* and OE-*TaMDHAR* lines under freezing stress. Mean ± SD (n = 3) is used to represent values. Two-way ANOVA was used to calculate significant differences, which are marked by asterisks; *, *p* < 0.05; **, *p* < 0.01; ***, *p* < 0.001; ****, *p* < 0.0001.

To further explore whether ROS scavenging was dependent on the AsA–GSH cycle in the OE-*TaMYB4* and OE-*TaMDHAR* lines, we identified the expression and activity of antioxidant enzymes related to the AsA–GSH cycle. The results showed that the OE-*TaMYB4* and OE-*TaMDHAR* lines had a higher expression of *AtSOD1*, *AtSOD2*, *AtAPX1*, *AtAPX6*, *AtDHAR1*, *AtDHAR2*, *AtGR1* and *AtGR2* than WT at 4 °C and −10 °C. OE-*TaMYB4* and OE-*TaMDHAR* lines also had higher SOD, APX, DHAR and GR activities than WT at 4 °C and −10 °C. At normal and low temperatures, OE-*TaMDHAR* lines had a higher MDHAR activity than WT (Figure 12 and Figure 13).

**Figure 14:**
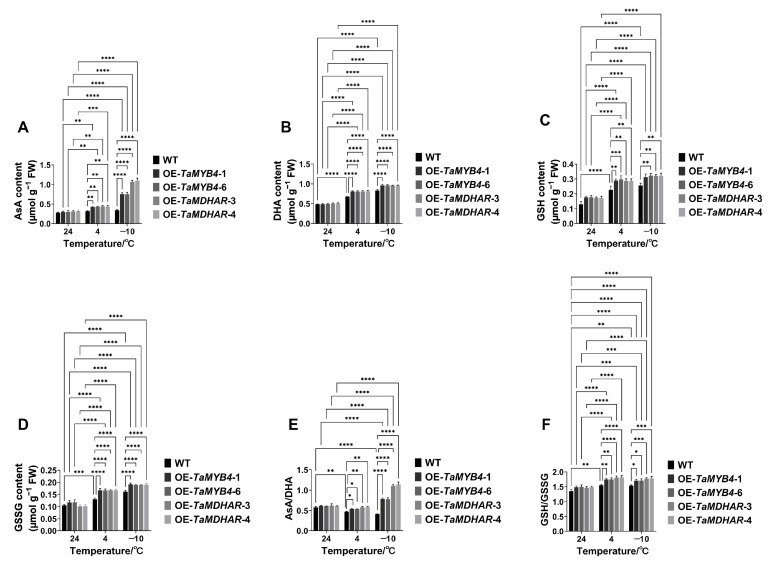
Metabolite contents of the AsA–GSH cycle in the OE-*TaMYB4* and OE-*TaMDHAR* lines under freezing stress. (**A**) AsA content; (**B**) DHA content; (**C**) GSH content; (**D**) GSSG content; (**E**) AsA/DHA; (**F**) GSH/GSSG. Mean ± SD (n = 3) is used to represent values. Two-way ANOVA was used to calculate significant differences, which are marked by asterisks; *, *p* < 0.05; **, *p* < 0.01; ***, *p* < 0.001; ****, *p* < 0.0001.

We determined the content of antioxidant substances in the OE-*TaMYB4* and OE-*TaMDHAR* lines. The results showed that the OE-*TaMYB4* and OE-*TaMDHAR* lines had higher AsA, DHA, GSH and GSSG contents than WT at 4 °C and −10 °C. The OE-*TaMYB4* and OE-*TaMDHAR* lines also had higher AsA/DHA and GSH/GSSG than WT at 4 °C and −10 °C (Figure 14).

### 2.8. Cold-Responsive Genes Expression Is Affected in OE-TaMYB4 Arabidopsis under Freezing Stress

**Figure 15:**
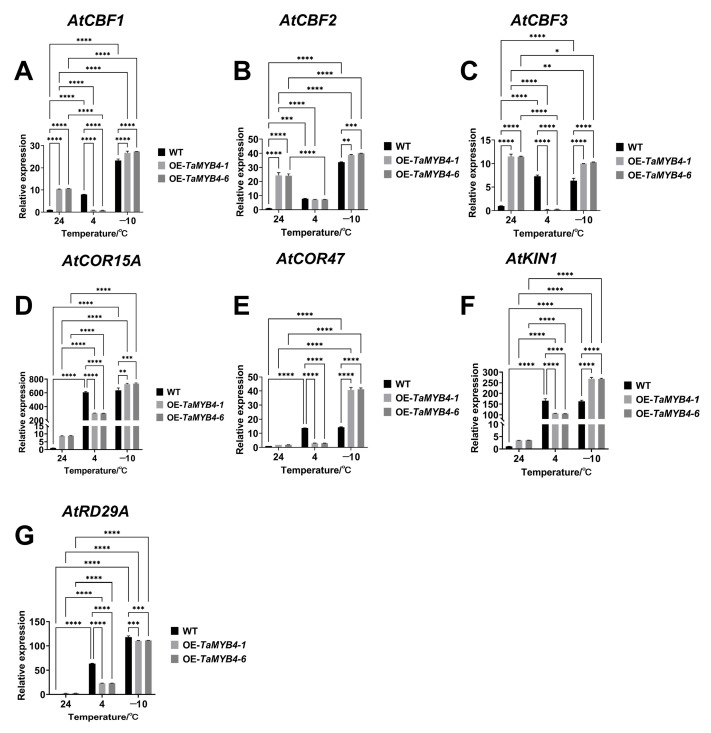
Expression of *AtCBF1* (**A**), *AtCBF2* (**B**), *AtCBF3* (**C**), *AtCOR15A* (**D**), *AtCOR47* (**E**), *AtKIN1* (**F**) and *AtRD29A* (**G**) in the OE-*TaMYB4* lines under freezing stress. Mean ± SD (n = 3) is used to represent values. Two-way ANOVA was used to calculate significant differences, which are marked by asterisks (24 °C WT as a control); *, *p* < 0.05; **, *p* < 0.01; ***, *p* < 0.001; ****, *p* < 0.0001.

*AtCBF1*, *AtCBF2*, *AtCBF3*, *AtCOR15a*, *AtCOR47*, *AtKIN1* and *AtRD29A* are typical cold stress marker genes [24]. We examined the expression of these genes in OE-*TaMYB4* lines. The results showed that the OE-*TaMYB4* lines had a higher expression of *AtCBF1*, *AtCBF2* and *AtCBF3* than WT at 24 °C and −10 °C. And the OE-*TaMYB4* lines had a higher expression of *AtCOR15a*, *AtCOR47*, *AtKIN1* and *AtRD29A* than WT at −10 °C. However, at 4 °C, the expression of these genes in OE-*TaMYB4* was not affected or significantly lower than those of WT (Figure 15).

## 3. Discussion

In multiple plants, MYB4 has been shown to be able to respond to varying degrees of cold stress. The *OsMYB4* expression was upregulated after 5 h of cold treatment (4 °C) [25]. The *LcMYB4* expression was consistently upregulated in *Leymus chinensis* within 24 h of cold treatment (4 °C) [26]. Under chilling (4 °C) and freezing stress (−4 °C), *MYB4* expression is significantly upregulated in different apple varieties [27,28]. After cold treatment at 4 °C for 2 h, *TaMYB4* expression is significantly upregulated in spring wheat [29]. However, the expression and function of *TaMYB4* in wheat under freezing stress are still unknown. The emergence of Dn1 provides a possibility to explore the freezing tolerance functions of wheat MYB TFs. In the Dn1 transcriptome, we found that several MYB TFs were differentially expressed under freezing stress. Among them, only the transcriptional level of *TaMYB4* was consistently upregulated with the decline in temperature (with the minimum temperature being −25 °C) [14]. In this research, *TaMYB4* expression was significantly upregulated in Dn1 under freezing stress (Figure 1A). Together with the findings of previous studies, this suggests that wheat TaMYB4 may function under both chilling and freezing stress. When the winter field temperature falls to −10 °C, the molecular, physiological and biochemical levels of Dn1 begin to change significantly [5,30,31]. In this research, *TaMYB4* expression was initiated at −10 °C in Dn1, functioning sooner than *TaMYB4* in J22. *TaMYB4* expression was significantly upregulated in field-grown J22 at −20 °C and −25 °C, but the fold change was significantly lower than that of Dn1 (Figure 1B). This suggests that Dn1 might initiate cold stress defense mechanisms sooner than J22. Our previous study effectively excluded the influence of temporal and spatial differences and other environmental factors on gene expression in Dn1 through short-term low-temperature treatment in the greenhouse [7]. In this research, the *TaMYB4* expression pattern in Dn1 under short-term low-temperature treatment in the greenhouse was similar to that of Dn1 in the field (Figure 1C). This suggests that *TaMYB4* functions under both long-term and short-term freezing stress, and that its expression may be mainly induced by freezing stress.

In plants, MYB TFs typically function by regulating downstream gene expression under cold stress. The regulatory mechanisms of AtMYB15 and AtMYB96 in *Arabidopsis* have been well characterized. MYB15 can either directly inhibit *CBF* expression or interact with ICE1 to regulate *CBF* expression [32]. MYB96 positively regulates *HHPs* expression under cold stress. HHP proteins subsequently activate *CBF* expression by interacting with ICE1 [10]. The above studies indicate that MYB TFs affect plant cold tolerance by regulating the CBF signaling pathway. However, it is rarely reported that MYB TFs directly regulate antioxidant enzyme genes. In this study, we had several lines of evidence suggesting that TaMYB4 directly regulates *TaMDHAR* expression in Dn1 under freezing stress. First, WGCNA revealed an association between *TaMYB4* and *TaMDHAR* (Figure 2A). Second, *TaMYB4* and *TaMDHAR* had similar expression patterns under freezing stress (Figure 1 and Figure 2). Third, TaMYB4 was found to be located in the nucleus and to possess transcription activation properties (Figure 3 and Figure 4). Finally, TaMYB4 was found to recognize and bind to five MYB binding sites in the *TaMDHAR* promoter (Figure 5, Figure 6 and Figure 7). Therefore, we speculate that TaMYB4 directly regulates *TaMDHAR* expression in Dn1 in response to freezing stress.

Extremely low temperatures not only cause freezing damage to plants but also result in the accumulation of intracellular ROS. If the antioxidant defense system fails to maintain the ROS level in a normal state, oxidative damage will occur. Excessive ROS leads to increased MDA content and electrical conductivity, which are biomarkers of cell membrane damage [33]. Overexpressing *OsMYB4* can reduce MDA content and electrical conductivity, thereby enhancing the cold tolerance of *Arabidopsis*, tomato and apple [25,34,35,36]. The overexpression of *pgMYB4* and *LcMYB4* can increase Pro content and confer stronger freezing tolerance to *Arabidopsis* [26,37]. In this study, the OE-*TaMYB4* lines had a higher Pro content and lower EC, MDA content and ROS content than WT under freezing stress (Figure 9 and Figure 11). These results coincide with those of previous studies and illustrate that TaMYB4 is able to improve plant freezing tolerance.

In plants, the antioxidant defense system consists of nonenzymatic and enzymatic antioxidant substances, which maintain the homeostasis of ROS. The enzymatic antioxidants comprise APX, MDHAR, DHAR, GR, GPX, PRX, CAT and SOD, while the nonenzymatic antioxidants comprise AsA, GSH, carotenoids, tocopherols, flavonoids, etc. Among them, AsA, GSH, APX, DHAR, MDHAR and GR constitute the well-known AsA–GSH cycle [38]. MDHAR is a momentous enzyme that maintains the reduced pool of AsA in this cycle [15]. In this study, we found that AtMDHAR2 shares the highest homology with TaMDHAR (Figure S3D) and contains MYB binding sites in its promoters (Figure S4). OE-*TaMYB4* lines had higher *AtMDHAR2* expression and MDHAR activity than WT under freezing stress (Figure 10). Therefore, we speculate that TaMYB4 can control endogenous *AtMDHAR2* expression, thereby enhancing the MDHAR activity of *Arabidopsis* under freezing stress. This suggests that the regulatory relationship between TaMYB4 and *MDHAR* may be conserved in plants. We found that overexpression of *TaMYB4* could promote the expression of antioxidant enzyme genes in the AsA–GSH cycle under freezing stress (Figure 12). In addition, OE-*TaMYB4* lines had higher antioxidant enzyme activities, AsA/DHA and GSH/GSSG in the AsA–GSH cycle than WT under freezing stress. These results were similar to the changes in physiological indices in the OE-*TaMDHAR* lines under freezing stress (Figure 13 and Figure 14). This suggests that TaMYB4 may regulate *MDHAR* expression to promote AsA–GSH cycle operation in *Arabidopsis*. Sun et al. found that the ectopic expression of *BrMDHAR* confers stronger freezing tolerance by inducing coregulation of the AsA–GSH cycle and enhancing the antioxidant capacity of host plants [39], which was consistent with our findings. The DREB/CBF signaling pathway plays an important role in plant cold resistance [24]. In this study, the OE-*TaMYB4* lines had a higher expression of *CBF1*, *CBF2*, *CBF3 COR15a*, *COR47*, *KIN1* and *RD29A* than WT at −10 °C. And the expression of *CBF1*, *CBF2* and *CBF3* in the OE-*TaMYB4* lines was also higher than those in WT at 24 °C (Figure 15). This result was not completely consistent with previous reports, indicating that TaMYB4 has specific regulation on these cold-responsive genes. However, the expression of these cold-responsive genes decreased in the OE-*TaMYB4* lines after 3 d of treatment at 4 °C. Previous reports have shown that the expression of these genes peaked after 3 or 24 h of treatment at 4 °C, and then gradually decreased [40,41]. This is consistent with our results. The suppression of the gene expression in the OE-*TaMYB4* lines at 4 °C may be to prevent the excessive defense of plants against chilling stress [42]. This suggests that TaMYB4 may affect the freezing tolerance of plants by regulating the expression of cold-responsive genes.

**Figure 16:**
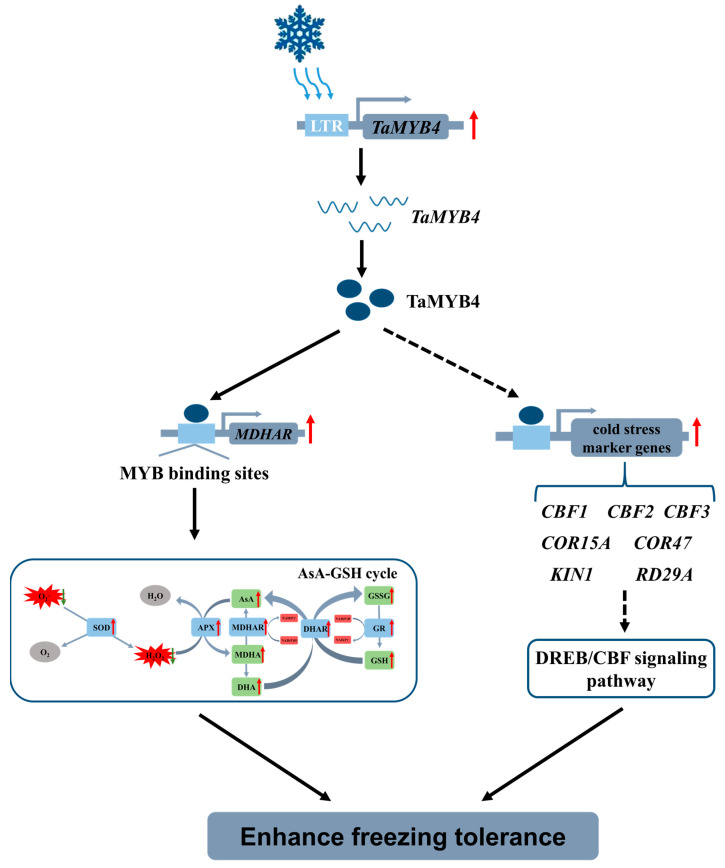
Potential mechanism model of TaMYB4 response to freezing stress in *Arabidopsis thaliana*.

In conclusion, the heterologous expression of *TaMYB4* promotes the AsA–GSH cycle operation and is associated with the upregulation of cold-responsive genes, thereby improving the freezing tolerance of transgenic *Arabidopsis* (Figure 16). Our findings on the functional role of *TaMYB4* will allow additional insight for developing freezing tolerant crops.

## 4. Materials and Methods

### 4.1. Plant Materials

The growing region and cultivation method of winter wheat referred to that used in previous studies [5]. Briefly, Dn1 and J22 (weakly cold-resistant variety) seeds were sown in the experimental field on 10 September 2019. When the average value of the lowest temperature of ten consecutive days reached 5 °C (1 October 2019), 0 °C (1 November 2019), −10 °C (18 November 2019) and −25 °C (31 December 2019), the tillering nodes were sampled. For the greenhouse component of the study, the winter wheat indoor cultivation method from previous studies was improved upon [7]. Briefly, Dn1 seedlings were cultivated in a 22 °C greenhouse (15 d) and subsequently transferred to a 5 °C incubation chamber for cold acclimation (30 d). Then, the temperature was gradually decreased and the seedlings were treated for 1 d each at 0, −10 and −25 °C. The tillering nodes of wheat were sampled at the above temperatures.

The *Arabidopsis* (Colombia ecotype) cultivation method was described in previous studies [4]. Briefly, *Arabidopsis* seedlings were cultivated in a greenhouse at 24 °C for 28 d and subsequently transferred to a 4 °C incubation chamber for 3 d of cold acclimation. After cold acclimation, the seedlings were subjected to 2 h of cold treatment at −10 °C. The leaves were sampled at 24, 4 and −10 °C. All plant materials mentioned above were stored at −80 °C. After 7 d of recovery culture, the survival rate was determined.

### 4.2. Quantitative Real-Time PCR

Total RNA was isolated from plants with TRIzol reagent (CWBIO, Beijing, China). The cDNA was subsequently obtained from mRNA with a HiScript III 1st Strand cDNA Synthesis Kit (Vazyme, Nanjing, China). Quantitative real-time PCR (qRT-PCR) was used for gene quantification analysis, and the detailed experimental methods referred to those used previous studies [6]. The data were analyzed by the 2^−ΔΔCT^ method.

### 4.3. Cloning and Bioinformatic Analysis

The coding sequences (CDS) of *TaMYB4* (TraesCS7D02G272400) and *TaMDHAR* (TraesCS7D02G277500) were queried in the wheat multiomics database (http://202.194.139.32/) (accessed on 1 September 2020) and amplified with specific primers (Table S1).

Subcellular localization prediction, homologous sequence alignment and phylogenetic analysis of TaMYB4 and TaMDHAR were performed by referring to the method of previous studies [4].

### 4.4. Subcellular Localization Analysis

The CDS of *TaMYB4* and *TaMDHAR* were constructed into the pCAMBIA2300-EGFP vector. Tobacco leaves (30 days old) were then injected with *Agrobacterium tumefaciens* GV3101 harboring pCAMBIA2300-EGFP, pCAMBIA2300-TaMYB4-EGFP or pCAMBIA2300-TaMDHAR-EGFP plasmids. After 72 h of injection, the leaves were harvested to observe the EGFP signal under a fluorescence microscope. Western blot analysis was performed to further analyze the subcellular localization results. The detailed experimental methods refer to those used by the authors in [43]. Anti-EGFP antibody (1:1000, Proteintech, Wuhan, China) was used as the primary antibody to probe EGFP. Horseradish peroxidase (HRP)-conjugated anti-rabbit antibody was chosen as the secondary antibody (1:2000, Proteintech).

### 4.5. Yeast One-Hybrid Assay

*TaMYB4* was constructed into the pGADT7 vector as an effector. There are five MYB binding sites (MRE, MBS, Myb, MYBrs and MYBbs) in the 2000 bp promoter sequence of *TaMDHAR*. Three tandem copies of MYB binding sites and the 2000 bp promoter sequence of *TaMDHAR* were separately constructed into the pHis2 vector as reporters. The protein–DNA interactions were analyzed according to the growth status of the Y187 strain containing reporter and effector on triple dropout synthetically defined (SD) medium lacking Leu, Trp and His containing 60 mM 3-AT.

### 4.6. Transient Expression Assay in Tobacco

To analyze the promoter activity and validate the regulatory relationship between TaMYB4 and *TaMDHAR*, a transient expression assay for tobacco (*Nicotiana tabacum*) was performed. The sequence of the *TaMYB4* promoter (2000 bp) was constructed into the pBI121-GUS vector. Tobacco leaves (30 days old) were then injected with *A. tumefaciens* GV3101 harboring the pBI121-ProTaMYB4-GUS plasmid. After 72 h of injection, the tobacco was subjected to 2 h of cold treatment at 4 °C. The leaves were collected for histochemical staining. Then, the GUS gene quantitative detection kit (Coolaber, Beijing, China) was applied for determining GUS activity in tobacco leaves.

The *TaMDHAR* promoter was truncated according to the MYB binding site distribution, with the 2000 bp promoter sequence and truncation sequences (P1, P2, P3 and P4) constructed into the pBI121-GUS vector as reporters. The *TaMYB4* overexpression construct was used as an effector. Tobacco leaves (30 days old) were then injected with *A. tumefaciens* GV3101 harboring the reporter and effector in a 1:1 ratio. After 72 h of injection, the tobacco was subjected to 2 h of cold treatment at 4 °C. The leaves were collected for histochemical staining and GUS activity assays.

The sequence of *TaMDHAR* promoter (2000 bp) and truncation sequences were also constructed into the pGreen II 0800-LUC vector. The TaMYB4 overexpression construct was used as an effector. Tobacco leaves (30 days old) were then injected with *A. tumefaciens* GV3101 (pSoup) harboring the reporter and effector in a 1:1 ratio. After 48 h of injection, the tobacco was subjected to 2 h of cold treatment at 4 °C. The LUC signal was visualized with a CCD system. Then, the Dual Luciferase Reporter Gene Assay Kit (Yeasen, Shanghai, China) was applied for determining LUC and REN activities in tobacco leaves.

### 4.7. Plant Overexpression Vector Construction and Transformation

The CDS of *TaMYB4* and *TaMDHAR* were constructed into the pCAMBIA2300-35S vector. Plasmids pCAMBIA2300-35S-TaMYB4 and pCAMBIA2300-35S-TaMDHAR were introduced into *A. tumefaciens* GV3101. The OE-*TaMYB4* and OE-*TaMDHAR* lines were generated by transforming pCAMBIA2300-35S-TaMYB4 and pCAMBIA2300-35S-TaMDHAR plasmids into *Arabidopsis* utilizing the floral dip method.

### 4.8. Physiological Indices Determination

The content of H_2_O_2_ in *Arabidopsis* leaves was assessed by DAB staining, and the content of O_2_^•−^ was assessed by NBT staining. The detailed experimental methods refer to those used by the authors in [44]. H_2_O_2_ and O_2_^•−^ contents were determined using a commercial assay kit (Comin, Suzhou, China), and the specific procedures were carried out as per the manufacturer’s instructions. Briefly, the H_2_O_2_ content was determined by monitoring the absorbance of the titanium–peroxide complex formed by H_2_O_2_ and titanium sulfate at 415 nm [45]. The O_2_^•−^, hydroxylamine hydrochloride, p-aminobenzenesulfonic acid and α- Naphthylamine reaction generates azo compounds. The O_2_^•−^ content was determined by monitoring the absorbance of azo compounds at 530 nm [46].

The MDA content and EC determination method was carried out in accordance with a previous study [47]. Pro content was determined using a kit (Comin). Briefly, the pro content was determined by monitoring the absorbance of the red substance formed by the reaction of proline and acid ninhydrin at 520 nm [48].

Antioxidant enzyme activity and non-enzymatic antioxidant content were also determined using kits (Comin), and the specific procedures were carried out in accordance with the manufacturer’s instructions. Briefly, SOD can remove the O_2_^•−^ in the reaction system. SOD activity was determined by monitoring the absorbance of blue formazan generated by the reaction of the remaining O_2_^•−^ with nitro-blue tetrazolium at 560 nm [49]. APX catalyzes the oxidation reaction of AsA and H_2_O_2_. The APX activity was calculated by measuring the AsA oxidation rate [50]. DHAR catalyzes the reduction reaction of GSH and DHA. DHAR activity was calculated by measuring the rate of DHA reduction [51]. MDHAR catalyzes the reduction reaction of NADPH and MDHA. MDHAR activity was determined by monitoring the absorbance of NADPH at 340 nm [52]. GR catalyzes the reduction reaction of NADPH and GSSG. GR activity was determined by monitoring the absorbance of NADPH at 340 nm [53].

The AsA content was determined by monitoring the absorbance of the substance generated by the reaction of AsA and o-Dianisidine bis (diazotized) zinc double salt at 420 nm [54]. Dithiothreitol reduces DHA to produce AsA. The DHA content was calculated by measuring the rate of AsA formation in the reaction system [55]. The GSH content was determined by monitoring the absorbance of the complex formed by the reaction of 5,5′-Dithiobis-(2-nitrobenzoic acid) and GSH at 412 nm. GR catalyzes the reduction of GSSG to generate GSH in the reaction system. The GSSG content was determined by monitoring the absorbance of the complex formed by the reaction of 5,5′-Dithiobis-(2-nitrobenzoic acid) and GSH at 412 nm [56]. The experimental data were calculated with fresh weight.

### 4.9. Statistical Analysis

Each experiment was performed with at least three biological replicates. All data, mean ± SD (standard deviation), were analyzed using the Student’s *t*-test and ANOVA (analysis of variance) with GraphPad Prism 9.0 at significance levels of * *p* < 0.05, ** *p* < 0.01, *** *p* < 0.001 and **** *p* < 0.0001.

## Data Availability

Data is contained within the article or Supplementary Material.

## Supplementary Materials

The supporting information can be downloaded at: https://www.mdpi.com/article/10.3390/ijms241311090/s1.

## References

[1] Wani S.H., Tripathi P., Zaid A., Challa G.S., Kumar A., Kumar V., Upadhyay J., Joshi R., Bhatt M. (2018). Transcriptional regulation of osmotic stress tolerance in wheat (*Triticum aestivum* L.). Plant Mol. Biol..

[2] Joshi R., Anwar K., Das P., Singla-Pareek S.L., Pareek A. (2017). Overview of Methods for Assessing Salinity and Drought Tolerance of Transgenic Wheat Lines. Methods Mol. Biol..

[3] Shi Y., Ding Y., Yang S. (2018). Molecular Regulation of CBF Signaling in Cold Acclimation. Trends Plant Sci..

[4] Tian Y., Peng K., Bao Y., Zhang D., Cang J. (2021). Glucose-6-phosphate dehydrogenase and 6-phosphogluconate dehydrogenase genes of winter wheat enhance the cold tolerance of transgenic *Arabidopsis*. Plant Physiol. Biochem..

[5] Bao Y., Xing J., Liang Y., Ren Z., Fu L., Yu J., Wang D., Zhang D., Xu Q., Cang J. (2022). Analysis of overwintering indexes of winter wheat in alpine regions and establishment of a cold resistance model. Field Crops Res..

[6] Peng K., Tian Y., Cang J., Yu J., Tan Y. (2022). Overexpression of TaFBA-A10 from Winter Wheat Enhances Freezing Tolerance in *Arabidopsis thaliana*. J. Plant Growth Regul..

[7] Peng K., Tian Y., Sun X., Song C., Ren Z., Bao Y., Xing J., Li Y., Xu Q., Yu J. (2021). tae-miR399-UBC24 Module Enhances Freezing Tolerance in Winter Wheat via a CBF Signaling Pathway. J. Agric. Food Chem..

[8] Wang X., Niu Y., Zheng Y. (2021). Multiple Functions of MYB Transcription Factors in Abiotic Stress Responses. Int. J. Mol. Sci..

[9] Wang Y., Mao Z., Jiang H., Zhang Z., Chen X. (2019). A feedback loop involving MdMYB108L and MdHY5 controls apple cold tolerance. Biochem. Biophys. Res. Commun..

[10] Lee H.G., Seo P.J. (2015). The MYB96-HHP module integrates cold and abscisic acid signaling to activate the CBF-COR pathway in *Arabidopsis*. Plant J..

[11] Li M., Lin L., Zhang Y., Sui N. (2019). ZmMYB31, a R2R3-MYB transcription factor in maize, positively regulates the expression of *CBF* genes and enhances resistance to chilling and oxidative stress. Mol. Biol. Rep..

[12] Park M.R., Yun K.Y., Mohanty B., Herath V., Xu F., Wijaya E., Bajic V.B., Yun S.J., De Los Reyes B.G. (2010). Supra-optimal expression of the cold-regulated OsMyb4 transcription factor in transgenic rice changes the complexity of transcriptional network with major effects on stress tolerance and panicle development. Plant Cell Environ..

[13] Zhang L., Zhao G., Jia J., Liu X., Kong X. (2012). Molecular characterization of 60 isolated wheat *MYB* genes and analysis of their expression during abiotic stress. J. Exp. Bot..

[14] Tian Y., Peng K., Lou G., Ren Z., Sun X., Wang Z., Xing J., Song C., Cang J. (2022). Transcriptome analysis of the winter wheat Dn1 in response to cold stress. BMC Plant Biol..

[15] Hasanuzzaman M., Hossain M.A., Silva J.A.T.D., Fujita M. (2012). Plant Response and Tolerance to Abiotic Oxidative Stress: Antioxidant Defense Is a Key Factor.

[16] El Airaj H., Gest N., Truffault V., Garchery C., Riqueau G., Gouble B., Page D., Stevens R. (2013). Decreased monodehydroascorbate reductase activity reduces tolerance to cold storage in tomato and affects fruit antioxidant levels. Postharvest Biol. Tec..

[17] Eltelib H.A., Badejo A.A., Fujikawa Y., Esaka M. (2011). Gene expression of monodehydroascorbate reductase and dehydroascorbate reductase during fruit ripening and in response to environmental stresses in acerola (*Malpighia glabra*). J. Plant Physiol..

[18] Park A.K., Kim I.-S., Do H., Kim H., Choi W., Jo S.-W., Shin S.C., Lee J.H., Yoon H.-S., Kim H.-W. (2019). Characterization and Structural Determination of Cold-Adapted Monodehydroascorbate Reductase, MDHAR, from the Antarctic Hairgrass *Deschampsia Antarctica*. Crystals.

[19] Shin S.Y., Kim I.S., Kim Y.S., Lee H., Yoon H.S. (2013). Ectopic expression of Brassica rapa L. MDHAR increased tolerance to freezing stress by enhancing antioxidant systems of host plants. S. Afr. J. Bot..

[20] Shin S.Y., Kim M.H., Kim Y.H., Park H.M., Yoon H.S. (2013). Co-expression of monodehydroascorbate reductase and dehydroascorbate reductase from *Brassica rapa* effectively confers tolerance to freezing-induced oxidative stress. Mol. Cells.

[21] Lou L., Li X., Chen J., Li Y., Tang Y., Lv J. (2018). Photosynthetic and ascorbate-glutathione metabolism in the flag leaves as compared to spikes under drought stress of winter wheat (*Triticum aestivum* L.). PLoS ONE.

[22] Khanna-Chopra R., Chauhan S. (2015). Wheat cultivars differing in heat tolerance show a differential response to oxidative stress during monocarpic senescence under high temperature stress. Protoplasma.

[23] Shan C., Ou X. (2018). Hydrogen peroxide is involved in the regulation of ascorbate and glutathione metabolism in wheat leaves under water stress. Cereal Res. Commun..

[24] Chen N., Pan L., Yang Z., Su M., Xu J., Jiang X., Yin X., Wang T., Wan F., Chi X. (2023). A MYB-related transcription factor from peanut, AhMYB30, improves freezing and salt stress tolerance in transgenic *Arabidopsis* through both DREB/CBF and ABA-signaling pathways. Front. Plant Sci..

[25] Vannini C., Locatelli F., Bracale M., Magnani E., Marsoni M., Osnato M., Mattana M., Baldoni E., Coraggio I. (2004). Overexpression of the rice *Osmyb4* gene increases chilling and freezing tolerance of *Arabidopsis thaliana* plants. Plant J..

[26] Li X.X., Jia J.T., Zhao P.C., Guo X.F., Chen S.Y., Qi D.M., Cheng L.Q., Liu G.S. (2020). LcMYB4, an unknown function transcription factor gene from sheepgrass, as a positive regulator of chilling and freezing tolerance in transgenic *Arabidopsis*. BMC Plant Biol..

[27] Wu R.G., Wang Y., Wu T., Xu X.F., Han Z.H. (2017). MdMYB4, an R2R3-Type MYB Transcription Factor, Plays a Crucial Role in Cold and Salt Stress in Apple Calli. J. Am. Soc. Hortic. Sci..

[28] Zhou P., Li X.S., Liu X.J., Wen X.J., Zhang Y., Zhang D.Y. (2021). Transcriptome profiling of Malus sieversii under freezing stress after being cold-acclimated. BMC Genom..

[29] Al-Attala M.N., Wang X., Abou-Attia M.A., Duan X., Kang Z. (2014). A novel TaMYB4 transcription factor involved in the defence response against *Puccinia striiformis* f. sp. tritici and abiotic stresses. Plant Mol. Biol..

[30] Lv Y., Song C.H., Lu Q.W., Tian Y., Li H.D., Zhang D., Yu J., Xu Q.H., Cang J. (2018). The Expression Characteristics of Transcription Factors Regulated by Exogenous ABA in Winter Wheat (*Triticum aestivum*) under Cold Stress. Russ. J. Plant Physiol..

[31] Lu Q., Xu Q., Guo F., Lv Y., Song C., Feng M., Yu J., Zhang D., Cang J. (2020). Identification and characterization of long non-coding RNAs as competing endogenous RNAs in the cold stress response of *Triticum aestivum*. Plant Biol..

[32] Agarwal M., Hao Y., Kapoor A., Dong C.H., Fujii H., Zheng X., Zhu J.K. (2006). A R2R3 type MYB transcription factor is involved in the cold regulation of *CBF* genes and in acquired freezing tolerance. J. Biol. Chem..

[33] Agarwal P., Mitra M., Banerjee S., Roy S. (2020). MYB4 transcription factor, a member of R2R3-subfamily of MYB domain protein, regulates cadmium tolerance via enhanced protection against oxidative damage and increases expression of *PCS1* and *MT1C* in *Arabidopsis*. Plant Sci..

[34] Pasquali G., Biricolti S., Locatelli F., Baldoni E., Mattana M. (2008). *Osmyb4* expression improves adaptive responses to drought and cold stress in transgenic apples. Plant Cell Rep..

[35] Vannini C., Campa M., Iriti M., Genga A., Faoro F., Carravieri S., Rotino G.L., Rossoni M., Spinardi A., Bracale M. (2007). Evaluation of transgenic tomato plants ectopically expressing the rice *Osmyb4* gene. Plant Sci..

[36] Mattana M., Biazzi E., Consonni R., Locatelli F., Vannini C., Provera S., Coraggio I. (2005). Overexpression of *Osmyb4* enhances compatible solute accumulation and increases stress tolerance of *Arabidopsis thaliana*. Physiol. Plant..

[37] Lian W.H., Sun T.X., Meng X.Y., Sun R., Hui F., Jiang Y.N., Zhao Y. (2021). Overexpression of the *Panax ginseng MYB4* gene enhances stress tolerance in transgenic *Arabidopsis thaliana*. Biol. Plant..

[38] Hasanuzzaman M., Bhuyan M., Anee T.I., Parvin K., Nahar K., Mahmud J.A., Fujita M. (2019). Regulation of Ascorbate-Glutathione Pathway in Mitigating Oxidative Damage in Plants under Abiotic Stress. Antioxidants.

[39] Zhang L.C., Liu G.X., Zhao G.Y., Xia C., Jia J.Z., Liu X., Kong X.Y. (2014). Characterization of a Wheat R2R3-MYB Transcription Factor Gene, *TaMYB19*, Involved in Enhanced Abiotic Stresses in *Arabidopsis*. Plant Cell Physiol..

[40] Kilian J., Whitehead D., Horak J., Wanke D., Weinl S., Batistic O., D’Angelo C., Bornberg-Bauer E., Kudla J., Harter K. (2007). The AtGenExpress global stress expression data set: Protocols, evaluation and model data analysis of UV-B light, drought and cold stress responses. Plant J..

[41] Zhou L., Yarra R., Yang Y., Liu Y., Yang M., Cao H. (2022). The oil palm R2R3-MYB subfamily genes *EgMYB111* and *EgMYB157* improve multiple abiotic stress tolerance in transgenic *Arabidopsis* plants. Plant Cell Rep..

[42] Novillo F., Alonso J.M., Ecker J.R., Salinas J. (2004). CBF2/DREB1C is a negative regulator of *CBF1/DREB1B* and *CBF3/DREB1A* expression and plays a central role in stress tolerance in *Arabidopsis*. Proc. Natl. Acad. Sci. USA.

[43] Deng X., Liu Y., Xu X., Liu D., Zhu G., Yan X., Wang Z., Yan Y. (2018). Comparative Proteome Analysis of Wheat Flag Leaves and Developing Grains Under Water Deficit. Front. Plant Sci..

[44] Dutilleul C., Garmier M., Noctor G., Mathieu C., Chétrit P., Foyer C.H., de Paepe R. (2003). Leaf mitochondria modulate whole cell redox homeostasis, set antioxidant capacity, and determine stress resistance through altered signaling and diurnal regulation. Plant Cell.

[45] Hussain H.A., Men S., Hussain S., Chen Y., Ali S., Zhang S., Zhang K., Li Y., Xu Q., Liao C. (2019). Interactive effects of drought and heat stresses on morpho-physiological attributes, yield, nutrient uptake and oxidative status in maize hybrids. Sci. Rep..

[46] Wang A.G., Luo G.H. (1990). Quantitative Relation between the Reaction of Hydroxylamine and Superoxide Anion Radicals in Plants. Plant Physiol. Commun..

[47] Wang R., Yu M., Xia J., Xing J., Fan X., Xu Q., Cang J., Zhang D. (2022). Overexpression of *TaMYC2* confers freeze tolerance by ICE-CBF-COR module in *Arabidopsis thaliana*. Front. Plant Sci..

[48] Chaoui A., Mazhoudi S., Ghorbal M.H., El Ferjani E. (1997). Cadmium and zinc induction of lipid peroxidation and effects on antioxidant enzyme activities in bean (*Phaseolus vulgaris* L.). Plant Sci..

[49] Huang J., Wang H., Zhong Y., Huang J., Fu X., Wang L., Teng W. (2019). Growth and physiological response of an endangered tree, Horsfieldia hainanensis merr., to simulated sulfuric and nitric acid rain in southern China. Plant Physiol. Biochem..

[50] Debnath B., Hussain M., Irshad M., Mitra S., Li M., Liu S., Qiu D. (2018). Exogenous Melatonin Mitigates Acid Rain Stress to Tomato Plants through Modulation of Leaf Ultrastructure, Photosynthesis and Antioxidant Potential. Molecules.

[51] Noctor G., Mhamdi A., Foyer C.H. (2016). Oxidative stress and antioxidative systems: Recipes for successful data collection and interpretation. Plant Cell Environ..

[52] Miyake C., Asada K. (1992). Thylakoid-Bound Ascorbate Peroxidase in Spinach Chloroplasts and Photoreduction of Its Primary Oxidation Product Monodehydroascorbate Radicals in Thylakoids. Plant Cell Physiol..

[53] Grace S.C., Logan B.A. (1996). Acclimation of Foliar Antioxidant Systems to Growth Irradiance in Three Broad-Leaved Evergreen Species. Plant Physiol..

[54] Yang Z., Cao S., Zheng Y., Jiang Y. (2012). Combined salicyclic acid and ultrasound treatments for reducing the chilling injury on peach fruit. J. Agric. Food Chem..

[55] Li F., Wu Q.Y., Duan M., Dong X.C., Li B., Meng Q.W. (2012). Transgenic tomato plants overexpressing chloroplastic monodehydroascorbate reductase are resistant to salt- and PEG-induced osmotic stress. Photosynthetica.

[56] Griffith O.W. (1980). Determination of glutathione and glutathione disulfide using glutathione reductase and 2-vinylpyridine. Anal. Biochem..

