# Supplementary material for: Overexpression of TaMYB4 Confers Freezing Tolerance in Arabidopsis thaliana
Source: Int J Mol Sci. 2023 Jul 4;24(13):11090. doi: 10.3390/ijms241311090 (PMC10342114; doi:10.3390/ijms241311090)
Supplement: Supplementary file 1 [file ijms-24-11090-s001.zip › Supplementary_Figures.pdf]

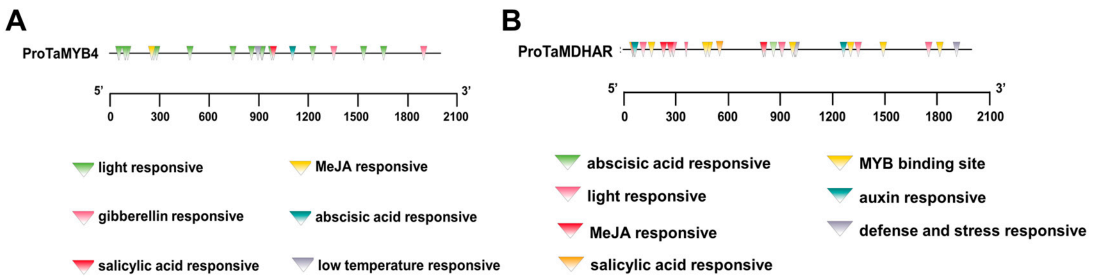

Supplementary Figure S1. ProTaMYB4 (A) and ProTaMDHAR (B) cis-acting element analysis.

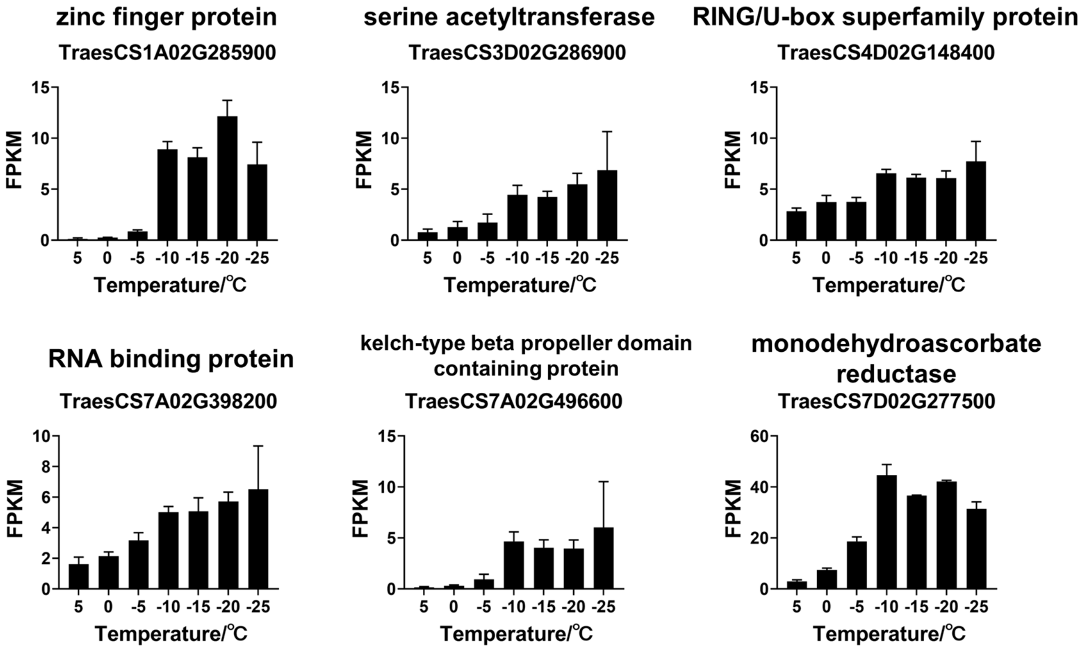

Supplementary Figure S2. Expression of *TaMYB4* associated genes in the Dn1 transcriptome.

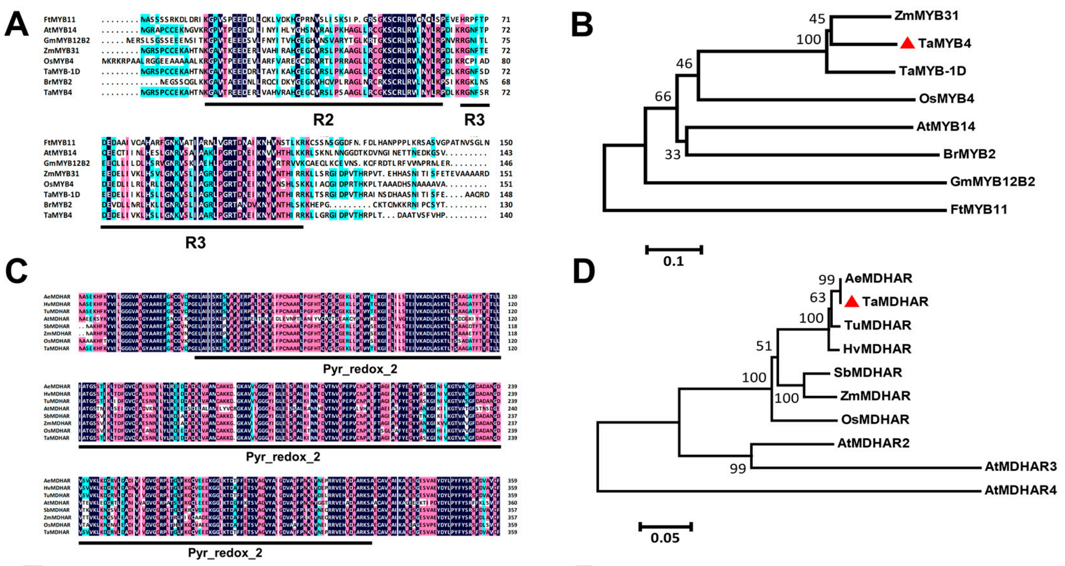

E

SeqID: TraesCS7D01G272400.1

Analysis Report:

SVM	LOCALIZATION	RELIABILITY
Amino Acid Comp.	Extracellular	0.752
N-peptide Comp.	Nuclear	0.465
Partitioned seq. Comp.	Nuclear	0.793
Physico-chemical Comp.	Nuclear	0.535
Neighboring seq. Comp.	Mitochondrial	0.550

CELLO Prediction:

Nuclear	1.957 *
Mitochondrial	1.340 *
Extracellular	1.161
Cytoplasmic	0.351
Peroxisomal	0.086
Chloroplast	0.055
Cytoskeletal	0.016
PlasmaMembrane	0.010
Golgi	0.007
Lysosomal	0.007
Vacuole	0.005
ER	0.005

F

SeqID: TraesCS7D01G277500.1

Analysis Report:

SVM	LOCALIZATION	RELIABILITY
Amino Acid Comp.	Cytoplasmic	0.686
N-peptide Comp.	Cytoplasmic	0.696
Partitioned seq. Comp.	Cytoplasmic	0.402
Physico-chemical Comp.	Chloroplast	0.367
Neighboring seq. Comp.	Chloroplast	0.608

CELLO Prediction:

Cytoplasmic	2.201 *
Chloroplast	1.503 *
ER	0.410
Mitochondrial	0.299
Peroxisomal	0.219
Vacuole	0.155
Golgi	0.054
Extracellular	0.048
Nuclear	0.048
PlasmaMembrane	0.042
Lysosomal	0.013
Cytoskeletal	0.007

Supplementary Figure S3. Bioinformatics analysis. (A) Alignment of homologous sequences of TaMYB4. (B) Phylogenetic tree of TaMYB4. (C) Alignment of homologous sequences of TaMDHAR. (D) Phylogenetic tree of TaMDHAR. (E) Prediction of subcellular localization of TaMYB4. (F) Prediction of subcellular localization of TaMDHAR.

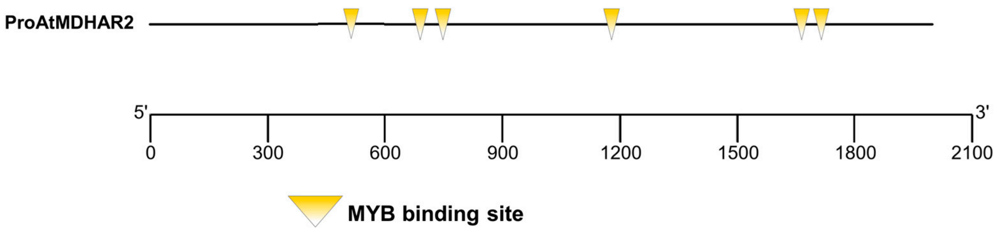

Supplementary Figure S4. Schematic diagram of 2000 bp ProAtMDHAR2. Cis-acting element analysis showed six MYB binding sites in ProAtMDHAR2, which are represented by yellow triangles.
